# Developing an implementation strategy for SBIRT in general hospital: first phase

**DOI:** 10.1186/1940-0640-10-S2-O27

**Published:** 2015-09-24

**Authors:** Alejandro Sánchez, Xóchitl de San Jorge

**Affiliations:** 1 Instituto de Ciencias de la Salud, Universidad Veracruzana, Xalapa, México

## Background

Following statement from Nilsen, Kaner and Babor[1], that motivation to address alcohol issues can be understood as a dynamic outcome between health professionals and patients embedded in the context, we follow a down-top implementation focus to work in partnership with all actors (professionals, patients and researchers) to understand ‘what’, ‘how’ and ‘why’ interventions can be carry out in everyday practice.

The objective is to develop an implementation strategy, grounded upon real conditions according Mexican healthcare settings in order to merge actors' needs encompassed into public health perspective. First phase's aims were: characterize actual practices oriented to reduce alcohol consumption over alcohol related disease inpatients, and to identify health professionals as key partners of implementation process.

## Material and methods

We use an Action-Research framework which is a reflective process of progressive problem solving led by individuals within everyday practice context[2]. Also an ethnographic approach was used to identify health professionals' activities around the healthcare of alcohol-related patients in one general hospital[3]. Forty-one interviews were conducted: 21 nurses, 9 medical practitioners, 11 social workers. Then three focus groups were set up to discuss interviewing findings.

## Results

Practitioners' activities are oriented to treat diseases, not persons. Younger practitioners showed concern over their biological focus. Referral to treatment is an uncommon practice. Nurses spend time talking to alcohol-related patients, giving advice about treatment after discharged and showed interest in understanding the patients' problems with alcohol. Social workers major focus is the financial support for medical treatment.

## Conclusions

In this first contact with the hospital context we have identified the nurses and younger medical practitioners as the key professionals to start joint projects (second phase). They recognize the limitation of their actions and the need for training in this field. Working close to them will let us identify “the conditions” for implementation strategies more than “barriers” to overcome.

## Consent to publish

Written informed consents were obtained from all interviewed health professionals who voluntary decided to participate the patient for publication of this exploratory study.
